# Morbidity Pattern Among Elderly in an Urban Area of Burla, Odisha

**DOI:** 10.7759/cureus.37189

**Published:** 2023-04-06

**Authors:** Pranab R Sahu, Subrat K Pradhan, Aruna Munda, Satish C Padhan, Ritesh R Behera, Linendra K Sahu, Sadhu C Panda

**Affiliations:** 1 Community Medicine, Veer Surendra Sai Institute of Medical Sciences and Research, Sambalpur, IND; 2 Community Medicine, Veer Surendra Sai Institute of Medical Science and Research, Sambalpur, IND

**Keywords:** cross sectional study, odisha, urban area, geriatric, morbidity pattern

## Abstract

Introduction: Population aging is an inevitable demographic reality that is associated with improvements in the health and medical care system. With longevity and declining fertility rates, the population of older persons is growing faster than the general population globally. The elderly population is more prone to various kinds of morbidity due to decreasing immunity and the risk of advancing age.

Objective: To describe the morbidity pattern of the elderly in an urban area of Burla.

Materials and methods: Community-based cross-sectional study was carried out for one year from 1st July 2021 to 30th June 2022. A total of 385 individuals aged 60 years and above residing in Burla were included in the study. Patient-wise data collection was done by a predesigned, pretested structured questionnaire. The chi-square test for categorical variables at a 95% confidence interval and significance set at 0.05 were used as measures of association in the analysis of factors associated with morbidity.

Result: The most common health problem involved was musculoskeletal (68.6%), followed by cardiovascular (57.1%), eye (47.3%), endocrine (25.2%), respiratory (21.3%), digestive (20.5%), skin (16.1%), ear (15.3%), general and unspecified health problems (30.7%), and urological (5.5%) and 4.5% had neurological problems.

Conclusion: Elderly population has a high frequency of numerous morbidities, so it is important to educate the elderly population about prevalent age-related health issues as well as preventive care.

## Introduction

People become increasingly susceptible to numerous diseases as they older, which is a normal biological process. The number and percentage of elderly persons in every country are rising with time. A correlation exists between improvements in the health and healthcare systems and the unavoidable demographic trend of population aging. Because of longer life expectancies and declining birth rates, the old population is growing more swiftly than the global population.

According to the 2011 Census, there are over 100 million senior citizens in India who are 60 years of age or older (8.6% of the total population) [1]. According to estimates, by 2050, it is estimated to reach more than 19% [2]. A total of 57 million disability-adjusted life years (DALYs), or 3.7% of all DALYs, are accounted for by this [3]. The proportion of the population over the age of 65 has increased from 6% in 1961 to 10.1% in 2021 and is expected to reach 13.1% in 2031. There seems to be a comparable trend in both urban and rural locations. Older people as a percentage of the population and vital statistics have risen in urban areas from 4.7% to 8.1% between 1961 and 2011, while they have increased in rural regions from 5.8% to 8.8%. Odisha would rank sixth among the states in the country by the end of 2031 for having a high percentage of senior people (Elderly India report). In 2011, there were 9.5% of elderly people in Odisha; by 2031, that number is expected to rise to 15.8%. According to the National Sample Survey Office, 45% of older people in rural areas and a comparable proportion in urban areas have chronic conditions. In contrast to urban areas, where hypertension, heart disease, and diabetes were more prevalent, rural areas had a higher prevalence of joint pain and cough. In both rural and urban locations, it was observed that 5% of the geriatric population was physically immobile [4].

According to figures provided by the Government of India, non-communicable diseases account for 52.5% of elderly mortality, followed by ill-defined disorders (25.7%), communicable diseases (18.1%), and injuries (3.7%) [5]. So, the purpose of this study was to describe the elderly's morbidity pattern in a Burla urban region.

## Materials and methods

A community-based cross-sectional study was carried out in the field practice area of the Urban Health Training Center, VIMSAR, Burla from 1st July 2021 to 30th June 2022. The study population comprised all elderly people of age ≥60 years, who have resided in the study area for at least one year. The total population of the field practice area of the urban health training center, VIMSAR, Burla, was 22,544 and out of which the geriatric population was 1670 (data compiled from Anganwadi center). Line listing of all the 1670 persons aged 60 years and older was done, and 385 such individuals were identified by systematic random sampling. Every fourth geriatric individual (like fourth, eighth, and 12th) was considered. Whenever the selected participant did not fulfill the inclusion criteria the next individual was selected. Data were collected till the desired sample size was achieved.

Inclusion criteria 

All individuals who were 1) 60 years or older, 2) staying more than one year in the research location, and 3) able to give informed consent were included.

Exclusion criteria

All study subjects who were 1) critically ill and who had 2) cognitive impairment were excluded. 

Sample size estimation

The sample size was calculated by using the formula n = Z_1-α/2_2P(1-P)/ d^2^, where n = sample size, p = prevalence of hypertension, d = allowable error, 5%, and Z1-α/22 = standard normal variate (p < 0.05) = 1.96. Considering p = 50%, the final sample size was calculated to be the minimum sample, n = 385. 

The elderly persons of the sampled population were interviewed by using predesigned and pretested proforma after taking informed consent. Information on selected sociodemographic characteristics, i.e. age, religion, types of family, socioeconomic status (SES), and morbidity pattern, was collected by taking history and examining the selected elderly person or from the existing medical records. 

Study tool

The Wonga International Classification Committee's (WICC), International Classification of Primary Care, 2nd Edition was used to classify the study participants' morbidity status in relation to the affected health system.

Data analysis

Analyses were carried out in MS Excel using proportion, percentage, and chi-square tests with p values <0.05 considered statistically significant.

Ethical clearance

The study was ethically approved by the Institutional Ethical Committee of the Veer Surendra Sai Medical College and Research (VIREC 057-2022/I-S-T/31/Dt.17.05.2022).

The protocol and importance of the study were explained to the participants before recruitment into the study, followed by a signed informed consent by them.

## Results

A total of 385 study participants were interviewed for the survey. All participants are urban dwellers. Out of these, 137 (35.6%) were male participants and 248 (64.4%) were female. The mean age of the study participants was 66.29 ± 7.01 years. The mean age of males in the population was 67.09 ± 8.26 years, while for females it was 65.84 ± 6.12 years. All members of the population belonged to the Hindu religion. Most females were homemakers (86.7%) and most males were laborers (48.4%) by occupation. The majority of respondents had not attended school (56.6%). Among them, most male respondents completed primary education (51.8%), while most females had not gone to school (73.3%). A joint family system was seen among the population interviewed to be the most common (88.8%), followed by the nuclear family (8.3%) (Table 1).

**Table 1:** Sociodemographic factors of study participants (n = 385)

Factors	Number (n = 385)	Percentage
Age (in years)		
60-69	282	73.3
70-79	81	21
≥80	22	5.7
Gender		
Male	137	35.6
Female	248	64.4
Caste		
General	23	6
Other backward classes (OBC)	259	67.3
SC	69	17.9
ST	34	8.8
Type of family		
Nuclear	32	8.4
Joint	342	88.8
Extended	11	2.8
Education		
Illiterate	218	56.6
Primary	122	31.7
Secondary	31	8.1
Higher	14	3.6
Occupation		
Retired employee	46	11.9
Self-employed	53	13.8
Homemaker	215	55.8
Laborer	71	18.4
Socioeconomic status		
Lower	10	2.6
Upper lower	207	53.8
Lower middle	168	43.6

Most individuals (54%) belonged to the upper lower category, followed by 43.6% in the lower middle category in the modified Kuppuswamy Socioeconomic Scale 2022 [6]. 

Out of 385 elderly people, 353 (91.7%) study participants had one or the other chronic morbidity. All elderly in the age group of 71-80 years (100%) and >80 years (100%) had chronic morbidity. 

Table 2 depicts the distribution of study participants according to various chronic morbid conditions. The most common diseases were diseases of the musculoskeletal system (68.6%). Other common diseases were those of the circulatory system (57.1%); 47.3% had suffered from diseases of the eye and adnexa; 25.2% had suffered from the endocrine, nutritional, and metabolic diseases; 21.3% had diseases of the respiratory system; 16.1% had diseases of skin the and subcutaneous tissue; and only 4.5% suffered from the diseases of the nervous system.

**Table 2:** Distribution of morbidity pattern of the study subjects based on ICD 10 classification of diseases

ICD 10 classification of diseases	Males (n = 137) (%)	Females (n = 248) (%)	Total (n = 385) (%)
Diseases of musculoskeletal system (M00-M99)	71 (51.8)	193 (77.8)	264 (68.6)
Diseases of circulatory system (I00-I99)	64 (46.7)	156 (62.9)	220 (57.1)
Diseases of the eye and adnexa (H00-H59)	52 (37.9)	130 (52.4)	182 (47.3)
Diseases of oral cavity and salivary glands (K00-K14)	39 (28.4)	71 (28.6)	110 (28.4)
Endocrine, nutritional, and metabolic diseases (E00-E90)	28 (20.4)	69 (27.8)	97 (25.2)
Diseases of respiratory system (J00-J99)	25 (18.2)	57 (22.9)	82 (21.3)
Diseases of digestive system (K00-K93)	18 (13.1)	61 (24.5)	79 (20.5)
Diseases of skin and subcutaneous tissue (L00-L99)	14 (10.2)	48 (19.3)	62 (16.1)
Diseases of the ear and mastoid process (H60-H95)	12 (8.7)	47 (18.9)	59 (15.3)
Diseases of genitourinary system (N00-N99)	9 (6.5)	12 (4.8)	21 (5.5)
Diseases of nervous system (G00-G99)	7 (5.1)	10 (4.03)	17 (4.5)

Table 3 shows that the diseases of the eye and adnexa, oral cavity and ear, digestive system, and skin and nervous system disorders increase with increasing age and are more common in individuals above 80 years of age compared to individuals in the age group of 60-69 years and 70-79 years. This difference was found to be statistically significant.

**Table 3:** Association between morbidity pattern of the study subjects and their age group

System involved	60-69 years (n = 282)	70-79 years (n = 81)	> 80 years (n = 22)	Total (n = 385)	X^2^ value	df	p-Value
Musculoskeletal system	185 (65.6)	62 (76.5)	17 (77.2)	264 (68.6)	4.314	2	0.115
Circulatory system	154 (54.6)	50 (61.7)	16 (72.7)	220 (57.1)	3.616	2	0.16
Eye and adnexa	107 (37.9)	56 (69.1)	19 (86.3)	182 (47.3)	38.86	2	<0.001
Oral cavity and salivary gland	49 (17.3)	48 (59.2)	13 (59)	110 (28.4)	64.74	2	<0.001
Endocrine, nutritional, and metabolic system	63 (22.3)	25 (30.8)	9 (40.9)	97 (25.2)	5.483	2	0.064
Respiratory system	56 (19.8)	18 (22.2)	8 (36.3)	82 (21.3)	3.369	2	0.185
Digestive system	40 (14.1)	24 (29.6)	15 (68.1)	79 (20.5)	41.7	2	<0.001
Skin and subcutaneous tissue	32 (11.3)	18 (22.2)	12 (54.5)	62 (16.1)	31.03	2	<0.001
Ear and mastoid process	23 (8.1)	21 (25.9)	15 (68.1)	59 (15.3)	65.55	2	<0.001
Genitourinary system	14 (4.9)	5 (6.1)	2 (9.1)	21 (5.5)	0.776	2	0.678
Nervous system	5 (1.7)	8 (9.8)	4 (18.1)	17 (4.5)	20.26	2	<0.001

## Discussion

The average age of study participants was 65.84 ± 6.17 years for female participants and 67.09 ± 8.25 years for male participants, respectively. This finding was consistent with the research by Biswas et al. [2] (67.14 ± 7.81 years) and Kapil et al. (the mean age of the males was 69.5 ± 7.4 years and the mean age for females was 67.8 ± 7.2 years). More people (56.7%) were discovered to be illiterate. A similar conclusion (52.5%) was published by Kapil et al. [7].

The WICC's International Classification of Primary Care, Second Edition, was used to determine the state of the study population's morbidity pattern by asking study participants about their chronic medical disorders. In the present study, out of 385 participants, 353 (91.7%) were having chronic morbidity. Usha P, et al. [8] presented similar findings. In comparison, Polisetty and Seepana [9] at Visakhapatnam recorded 99.6% morbidity among older people, whereas a lower prevalence (83.9%) was found in Tamilnadu by Piramanayagam A, et al. [10].

After categorizing the chronic morbid illnesses according to the affected health system, further investigation revealed that the musculoskeletal system accounted for 68.6% of all involvements. Other frequently affected health systems included the cardiovascular (57.1%), eye (47.3%), endocrine (25.2%), respiratory (21.3%), digestive (20.5%), skin (16.1%), ear (15.3%), and urological (5.5%) systems. Very few elderly people reported neurological (4.5%) issues. The most prevalent cardiovascular illness was hypertension (56%). 

According to study findings, there are similar rates of musculoskeletal problems (71.4%), ocular and adnexal disorders (49.7%), oral cavity disorders (32.9%), endocrine, nutritional, and metabolic disorders (32.9%), and so on, to those identified in a study by Dhar et al. [11]. Involvement in the musculoskeletal system (68.5%) was the most prevalent, according to a study carried out by Verma V, et al. [12] in Allahabad. The following health systems were also frequently involved: psychological (59.75%), digestive (29.75%), ear (13%), and respiratory (11.25%). In a study conducted by Chauhan and Chandrashekar [13] at Nellore among elderly persons, it was discovered that the musculoskeletal (69.7%), digestive (16.2%), cardiovascular (38.3%), respiratory (26.9%), neurological (6.2%), psychological (12.8%), and urogenital (5.7%) systems were all affected. Cardiovascular disorders were also shown to be prevalent (31.70%) in a study conducted in Bhubaneswar by Mullick TH, et al. [14].

In our study, we found that older people had much higher rates of abnormalities of the eyes and adnexa, digestive system, circulatory system, and nervous system, than younger people. Pathak G et al. [15] and Shraddha et al. [16] similarly observed that there was a substantial increase in morbidity prevalence with age.

Limitation 

The study cannot examine the causal linkages because the data are cross-sectional. Self-reporting of chronic conditions raises the possibility of recall bias and prejudice in reporting among the elderly. The study can only be generalized to urban areas. Non-inclusion of the critically ill and cognitively impaired participant is also a limitation.

## Conclusions

Our study shows that the old population is highly susceptible to a variety of morbidities; hence, it is crucial to inform the senior population about common age-related health problems as well as preventive care. Government and non-governmental organizations should regularly hold geriatric screening activities to encourage a healthy, longer life and reduce morbidity through early diagnosis and treatment (secondary prevention).
